# The plant natural product 2-methoxy-1,4-naphthoquinone stimulates therapeutic neural repair properties of olfactory ensheathing cells

**DOI:** 10.1038/s41598-020-57793-2

**Published:** 2020-01-22

**Authors:** M. Chen, M. L. Vial, L. Gee, R. A. Davis, J. A. St John, J. A. K. Ekberg

**Affiliations:** 1 Clem Jones Centre for Neurobiology and Stem Cell Research, Griffith University, Nathan, 4111 QLD Australia; 2 Griffith Institute for Drug Discovery, Griffith University, Nathan, QLD 4111 Australia; 3 Menzies Health Institute Queensland, Griffith University, Southport, QLD 4222 Australia

**Keywords:** Drug screening, Cell division, Cellular imaging, Cell biology, Drug discovery, Cellular neuroscience, Glial biology, Olfactory system, Regeneration and repair in the nervous system

## Abstract

Olfactory ensheathing cells (OECs) are crucial for promoting the regeneration of the primary olfactory nervous system that occurs throughout life. Transplantation of OECs has emerged as a promising therapy for nervous system injuries, in particular for spinal cord injury repair. Functional outcomes in both animals and humans are, however, highly variable, primarily because it is difficult to rapidly obtain enough OECs for transplantation. Compounds which can stimulate OEC proliferation without changing the phenotype of the cells are therefore highly sought after. Additionally, compounds which can stimulate favourable cell behaviours such as migration and phagocytic activity are desirable. We conducted a medium-throughput screen testing the Davis open access natural product-based library (472 compounds) and subsequently identified the known plant natural product 2-methoxy-1,4-naphthoquinone as a stimulant of OEC viability. We showed that 2-methoxy-1,4-naphthoquinone: (i) strongly stimulates proliferation over several weeks in culture whilst maintaining the OEC phenotype; (ii) stimulates the phagocytic activity of OECs, and (iii) modulates the cell cycle. We also identified the transcription factor Nrf2 as the compound’s potential molecular target. From these extensive investigations we conclude that 2-methoxy-1,4-naphthoquinone may enhance the therapeutic potential of OECs by stimulating proliferation prior to transplantation.

## Introduction

The primary olfactory nervous system undergoes regeneration throughout life. The glial cells of this system, olfactory ensheathing cells (OECs), are thought to be critical for the regeneration. OECs provide physical and neurotrophic support^1–3^, and guide primary olfactory axons towards their targets in the olfactory bulb in the central nervous system (CNS)^4–7^. OECs have become promising candidates for cell transplantation therapies to repair CNS injuries, particularly spinal cord injury (SCI). Functional outcomes are, however, highly variable in both animal studies and human clinical trials and improvements are needed^8–12^. OECs have poor survival rate after transplantation and harvesting and generating enough cells for transplantation in a relatively short time is challenging^9^. Thus, small molecules that can promote OEC proliferation without loss of the cellular phenotype could enhance the therapeutic potential of OEC transplantation. We have previously shown that natural compounds can stimulate OEC viability. These include curcumin from turmeric^13^, linckosides from starfish^14^ and two serrulatane diterpenoids from the Australian desert plant *Eremophila microtheca*^15^.

Compounds capable of enhancing key OEC functions crucial for neural repair, such as migration and phagocytic activity, are also desirable^16,17^. OECs migrate ahead of axons^18,19^ and, after injury to the olfactory bulb, migrate from the periphery to enter the injury site in the CNS^20^. Some studies show that transplanted OECs exhibit strong capacity for migration into the injury site^21,22^, but others show that OEC migration is relatively limited^23^; thus, promoting OEC migration may lead to better nerve repair. Another important function of OECs is phagocytosis of cell debris. OECs are the primary phagocytic cells in the olfactory nerve during normal olfactory neuron turnover and after injury^24,25^. Transplanted OECs are capable of phagocytosing debris in the SCI site^26^. As debris removal is critical for regeneration, stimulation of OEC phagocytosis may also be favourable. Low-dose curcumin has been shown to strongly stimulate the phagocytic activity of OECs^13,27^, in addition to stimulating both proliferation and migration^13^. *Eremophila microtheca* compounds have also been shown to enhance phagocytic activity, migration and cell viability of OECs^15^. These findings show that it is possible to stimulate OEC functions that are important for neural repair.

To identify more compounds capable of stimulating OECs, we first conducted a medium throughput screen in which we tested the Davis open access natural product-based library (472 compounds)^28^ for enhancement of OEC viability and subsequently identified 2-methoxy-1,4-naphthoquinone (which has the Davis compound code RAD618) as a hit compound. 2-methoxy-1,4-naphthoquinone is a known plant natural product, which has previously been isolated from *Impatiens balsamina*^29^ and *Impatiens glandulifera*^30^. We then performed more detailed studies to investigate how this compound affects the cell cycle, migration and phagocytic activity of OECs.

## Results

### RAD618 stimulates OEC viability

**Figure 1:**
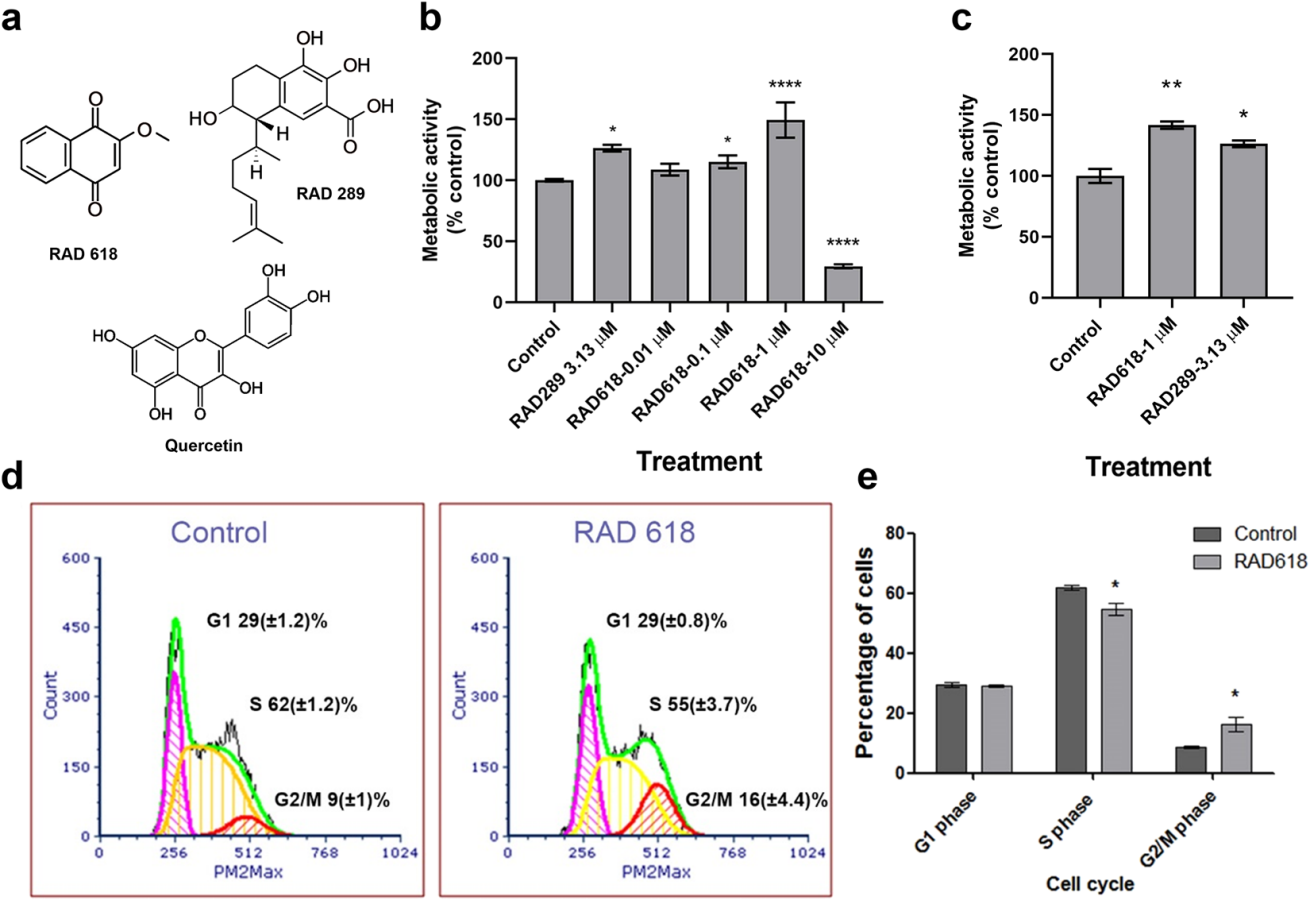
Effects of RAD618 (2-methoxy-1,4-naphthoquinone) on the metabolic activity and cell cycle of GFP-mOECs. (**a**) The chemical structure of RAD618 (2-methoxy-1,4-naphthoquinone) RAD289 (3,7,8-trihydroxyserrulat-14-en-19-oic acid) and quercetin. (**b**) The metabolic activity of mOECs exposed to RAD618 (0.01–10 µM) or in vehicle control (medium only) was determined by the resazurin metabolic activity indicator (viable cells metabolize resazurin to fluorescent resofurin; resofurin fluorescence is measured). Bars show the metabolic activity of RAD618-treated cells normalized to vehicle control in percentages. Triplicate wells were used in two separate experiments. Data are shown as mean ± SEM. *p < 0.05, ****p < 0.0001, one-way ANOVA, Tukey’s multiple comparison test. (**c**) Effects of RAD618 and RAD289 (positive control) on the metabolic activity of GFP-mOECs. For each treatment, 3 × 1000 cells/group with 2 technical repeats, were tested, data are shown as mean ± SEM. *p < 0.05, **p < 0.01, one-way ANOVA, Tukey’s multiple comparison test. (**d**) Cell cycle flow cytometry analysis of mOEC cultured in control medium and in medium containing 1 µM RAD618. Cells were gated via forwarding scatter and PI intensity (PM2max). Data presented is from a representative sort (based on three independent experiments with similar results). Pink areas show the percentages of cells in the G1 phase; yellow areas show percentages of cells in the S phase, and the red area show percentages of cells in the G2/M phase; green line, data density. (**e**) Summarized data of the cell cycle analysis (n = 3 repeats x 100,000 cells). Bars show the percentages of cells in the three different phases in the presence and absence of RAD618. Error bars show mean ± SEM. *p < 0.05, Student’s t-test.

Compounds that can enhance OEC viability, as well as cellular functions associated with neural repair, are likely to increase the therapeutic potential of OEC transplantation. We screened a library of 472 pure natural products or derivatives for the ability to promote OEC viability. Immortalized mouse OECs expressing GFP (GFP-mOECs) were cultured in 384-well plates (2000 cells per well). Cells were incubated with the natural products at 0.01 µM, 0.1 µM, 1 µM and 10 µM for 24 h. The metabolic activity indicator resazurin was used to measure viability (which can indicate increased proliferation and/or increased cell survival). After the first screening, natural products or compounds that increased cell viability more than 20% in relation to control (to ≥ 120%) were tested again in the same manner. In both screenings, one compound belonging to the naphthoquinone family, 2-methoxy-1,4-naphthoquinone and termed RAD618 in our library (Fig. 1a), was found to enhance cell viability at 0.1 and 1 µM. The effect was most pronounced at 1 µM (↑44% compared to control) (Fig. 1b). At 10 µM, RAD618 caused a pronounced decrease in cell viability; thus, the compound appeared cytotoxic at this concentration. We have previously identified that two serrulatane diterpenoid natural products, RAD288 (3-acetoxy-7,8-dihydroxyserrulat-14-en-19-oic acid) and RAD289 (3,7,8-trihydroxyserrulat-14-en-19-oic, Fig. 1a), stimulate viability of OECs, in particular RAD289^15^. We therefore used RAD289 as a positive control and tested RAD289 and RAD618 using the same viability assay which confirmed that RAD618 strongly stimulated the cell viability (Fig. 1c).

The fact that RAD618 stimulated proliferation at low concentrations indicated that the compound was worthy of further investigations.

### RAD618 induces cell cycle alterations in GFP-mOECs consistent with increased proliferation

To determine whether RAD618 stimulated proliferation by modulating the cell cycle, GFP-mOECs were treated with 1 µM RAD618 for 24 h and then labelled with the DNA-binding fluorescent dye propidium iodide (PI). The fluorescence intensity of the cells correlates with the DNA amount per cell, which differs in the G1, S or G2/M phase^31^. Flow cytometry analysis based on fluorescence intensity/cell was used to separate the three populations. In the control condition (0.2% DMSO), 29.37 ± 1.23% of cells were in the G1 phase, 61.95 ± 1.24% were in the S phase and 8.68 ± 0.95% were in the G2/M phase (Fig. 1d). The percentage for the RAD618 treatment group were as follow: G1 phase: 29.03 ± 0.82%, S phase: 54.71 ± 3.7% and G2/M phase: 16.26 ± 4.4% (Fig. 1c). Thus, RAD618 significantly decreased the percentage of cells in the S phase (by 7.24%; p < 0.05) and increased the percentage of cells in the G2/M phase (by 7.58%; p < 0.05) (Fig. 1e). The distribution of cells in the G1 phase and G2/M phase were not significantly affected by RAD618. Thus, RAD618 induced a shift from the S phase to the G2/M phase in the cell cycle.

**Figure 2:**
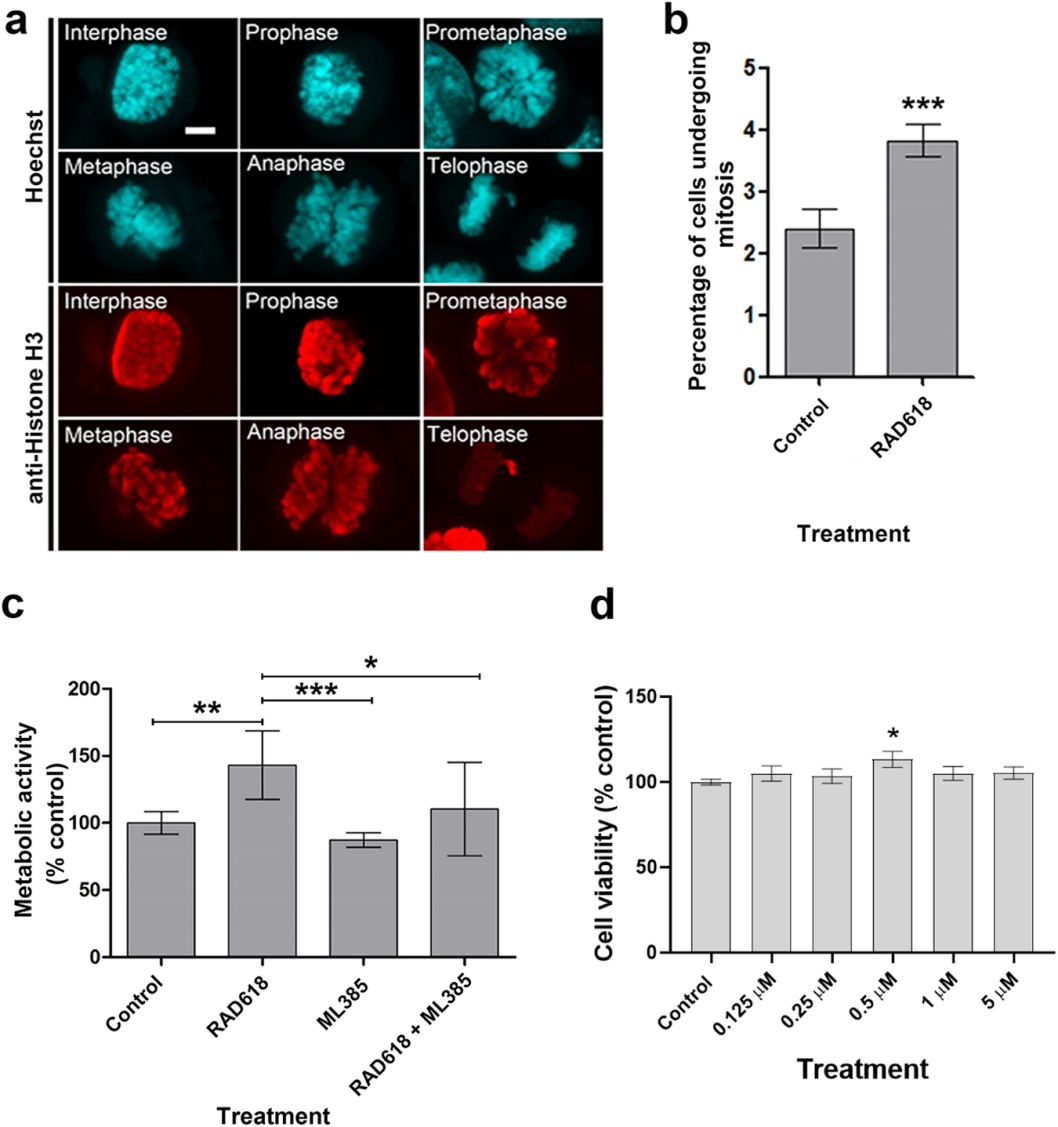
RAD618 induces mitosis of GFP-mOECs, and Nrf2 is a potential molecular target. (**a**) mOECs were stained with Hoechst (DNA stain, turquoise) and immunolabelled with an antibody directed against phosphor-Ser10 of Histone H3 (red). Histone H3 phosphorylation at Ser 10 was apparent during mitosis (interphase to anaphase) with distinctly different intensity and distribution of chromatin in the different mitotic phases. Scale bar: 2 µm. (**b**) Percentages of cells in the early stages of mitosis (including interphase, prophase, prometaphase, metaphase and anaphase) in cells incubated in vehicle control (cell medium only) and in medium with 1 µM RAD618. Triplicate wells were used in three separate experiments. Error bars show mean ± SEM. ***p < 0.001, Student’s t-test. (**c**) Inhibition of Nrf2 neutralized the effect of RAD618 on cell viability. GFP-mOECs were treated with culture medium only (vehicle control), RAD618 (1 µM), ML385 (5 µM), or RAD618 (1 µM) + ML 385 (5 µM). Cell viability was assessed with resazurin assay (n = 3 repeats). (**d**) The Nrf2 agonist quercetin stimulates the metabolic activity of OECs (n = 3 × 1000 cells/group, 2 technical repeats). Error bars show mean ± SEM. *p < 0.05, **p < 0.01, ***p < 0.001, ANOVA, Tukey’s multiple comparison test.

To further analyze the effect of RAD618 on the cell cycle, we then determined the specific effects of the compound on the different stages of mitosis. The stages of mitosis can be distinguished using specific immunolabelling for histone H3 phosphorylation at serine 10 (Ser10). Phosphorylation at Ser10 correlates with chromosome condensation, and thus the intensity and appearance of phospho-S10 histone H3 labelling is strikingly different in the different mitotic stages. Phosphorylation of Ser10 begins in the interphase, appears as an intact sphere in prophase, which is then separated during mitosis as the chromosomes separate, and disappears in the telophase^32^. GFP-mOECs were immunolabelled for α-phospho-S10-histone H3 after mOECs had been treated with RAD618 or control medium for 24 h. In GFP-mOECs, the intensity of anti- phospho-S10-histone H3 labelling was easily detected in the interphase, prophase, prometaphase, metaphase and anaphase, allowing clear distinction of the mitosis stages (Fig. 2a). We determined the percentages of cells in the early stages of mitosis (interphase to anaphase) in the absence and presence of RAD618. Significantly more cells in the early mitotic stages were present in the RAD618 group than in the control group (Fig. 2b, p < 0.001). Thus, our findings show that RAD618 stimulates mitosis. This in turn suggests that RAD618 enhances cell viability by stimulating proliferation.

### The transcription factor Nrf2 is a potential target of RAD618

Other naphthoquinones have been shown to stimulate the activity of nuclear factor (erythroid-derived 2)-like 2(Nrf2) in other cell types^33,34^. Nrf2 regulates cell cycle transition from G2 to M phase^35^. We thus hypothesised that RAD618 may act on Nrf2 because our data showed that RAD618 stimulated cell viability by increasing the number of cells undergoing mitosis, consistent with as Nrf2 activation (crucial for G2 to M phase transition and thus mitosis). We tested the effects of RAD618 on cell proliferation in the absence and presence of the Nrf2 inhibitor ML385, which blocks the DNA-binding site of Nrf2^36^. GFP-mOECs were treated by ML385 for 48 h before adding RAD618 or control medium. Then, the cell viability of the four groups (1. control, 2. RAD618 alone, 3. ML385 alone and 4. RAD618 + ML385) was measured by resazurin assay. ML385 was found to attenuate the effect of RAD618 on viability of GFP-mOECs (Fig. 2c). Therefore, Nrf2 is a potential target of RAD618. To confirm that stimulation of Nrf2 enhanced the metabolic activity of OECs, we also tested whether another known Nrf2 agonist, quercetin^37–39^ (Fig. 1a), had this effect. We found that quercetin at 0.5 µM stimulated the metabolic activity by 13% (Fig. 2d).

### Cell proliferation of primary mouse OECs is enhanced by RAD618 in long-term cultures

**Figure 3:**
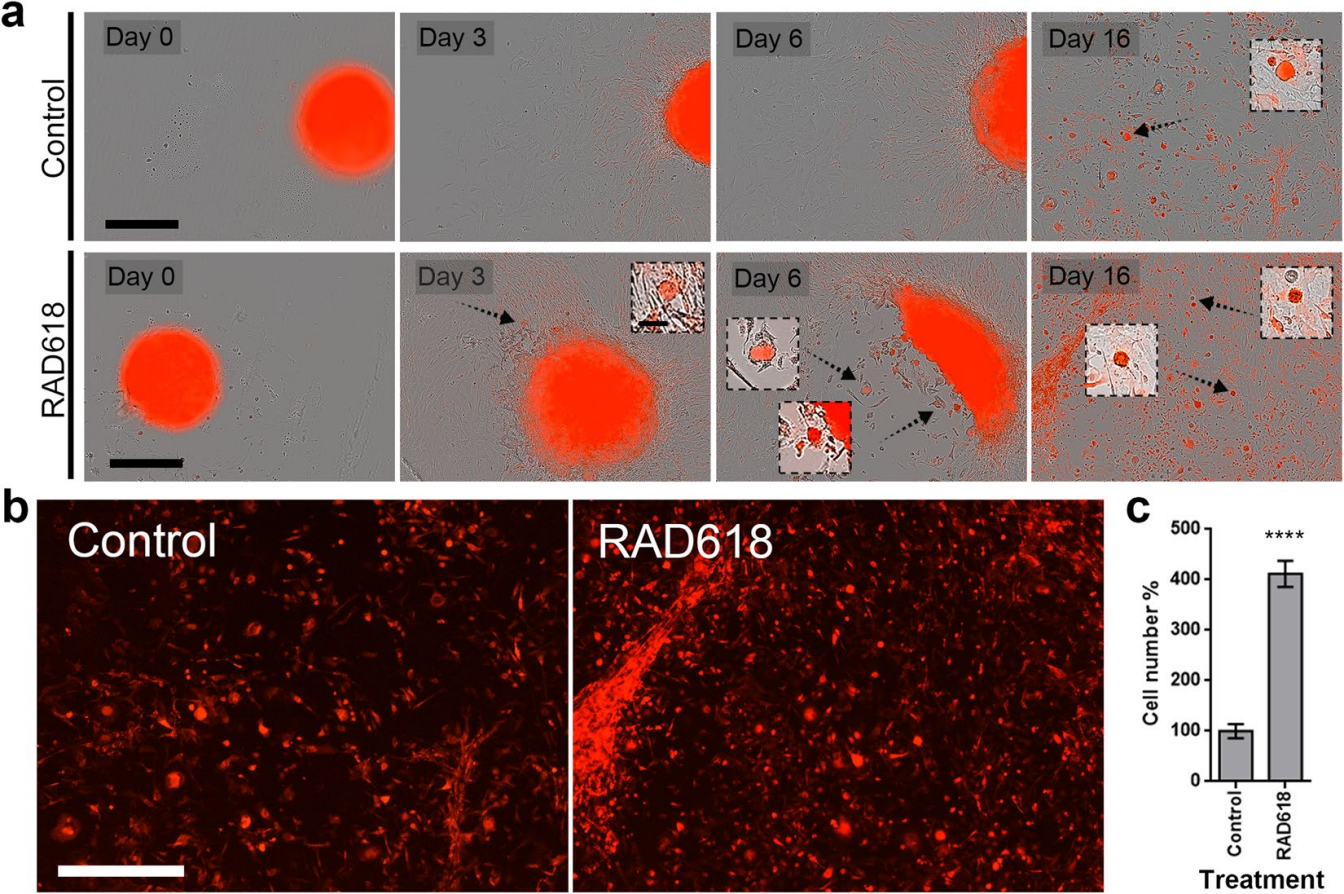
Cell proliferation of primary mouse OECs is enhanced by RAD618. Primary OECs were purified by a combination method of 3D cell culture and laser microdissection to reduce contaminating cells. After purification, cells were incubated in medium containing 2 µM RAD618 or in control medium without RAD618. Cells were imaged using an IncuCyte Live Cell Analysis Imaging System. (**a**) Images of live cells (bright field and red fluorescence overlay) in control medium and with RAD618 treatment were captured at day 0, day 3, day 6 and day 16. OECs but not contaminating cells exhibit red fluorescence. Scale bar: 400 µm. Small cell spheroids were present when cells reached higher density (arrows). Scale bar: 400 µm. (**b**) Fluorescence images (showing only DsRed-expressing OECs) of cells in control medium or RAD618-containing medium at day 16 in culture. Scale bar: 15 µm. (**c**) After 55 days, the cell numbers in control and under RAD618 treatments, were counted by Countess II FL Automated Cell Counter and Hemocytometer. n = 3 repeats, mean ± SEM. ****p < 0.0001.

For a compound to be therapeutically useful for stimulating OEC proliferation for transplantation purposes, it must act on primary OECs over an extended period in culture. We tested RAD618 in long-term assays on OECs isolated from the primary olfactory nervous system (lamina propria) of S100ß-DsRed transgenic mice, in which OECs express DsRed. The primary OEC culture was often contaminated with DsRed(−) cells, as previously shown^18^. OEC cultures were enriched using a combination method of Naked Liquid Marble (NLM) 3D culture^40^ and laser microdissection. OECs were allowed to form spheroids within the NLM (200,000 cells per spheroid), which were then transferred to a chamber in the absence or presence of RAD618 (Fig. 3). DsRed(−) cells migrated out of the spheroids ahead of OECs and could therefore be selectively ablated using laser microdissection (at 24, 32 and 40 h; Supp. Figs. 1 and 2). Cells were then allowed to continue migrating out of the spheroids over 16 days (Fig. 3a). The primary OECs spontaneously formed small cell spheroids in the culture. This occurred from day 3–6 onwards in the RAD618-treated group and from day 16 in the control group (Fig. 3a). After 55 days, cells were detached and counted. There were significantly more cells (4.1-fold increase) in the RAD618-treated group than in the control group (Fig. 3c).

### Primary OECs treated with RAD618 continue to express the p75 neurotrophin receptor in long-term cultures

Lamina propria-derived OECs express high levels of the p75 neurotrophin receptor (p75NTR)^19,41^. To ensure that the OEC phenotype was maintained, it is important that OECs do not de-differentiate in culture; this includes, in addition to continuous expression of S100β-driven DsRed, expression of p75NTR. A previous study showed that expression of p75NTR was reduced over time in long-time cultures of porcine OECs (strongly reduced after ~4 weeks). This correlated with poor OEC proliferation and loss of myelination capacity^42^ (OECs do not myelinate axons in their natural environment, but has the capacity to do so under certain conditions^43–45^).

**Figure 4:**
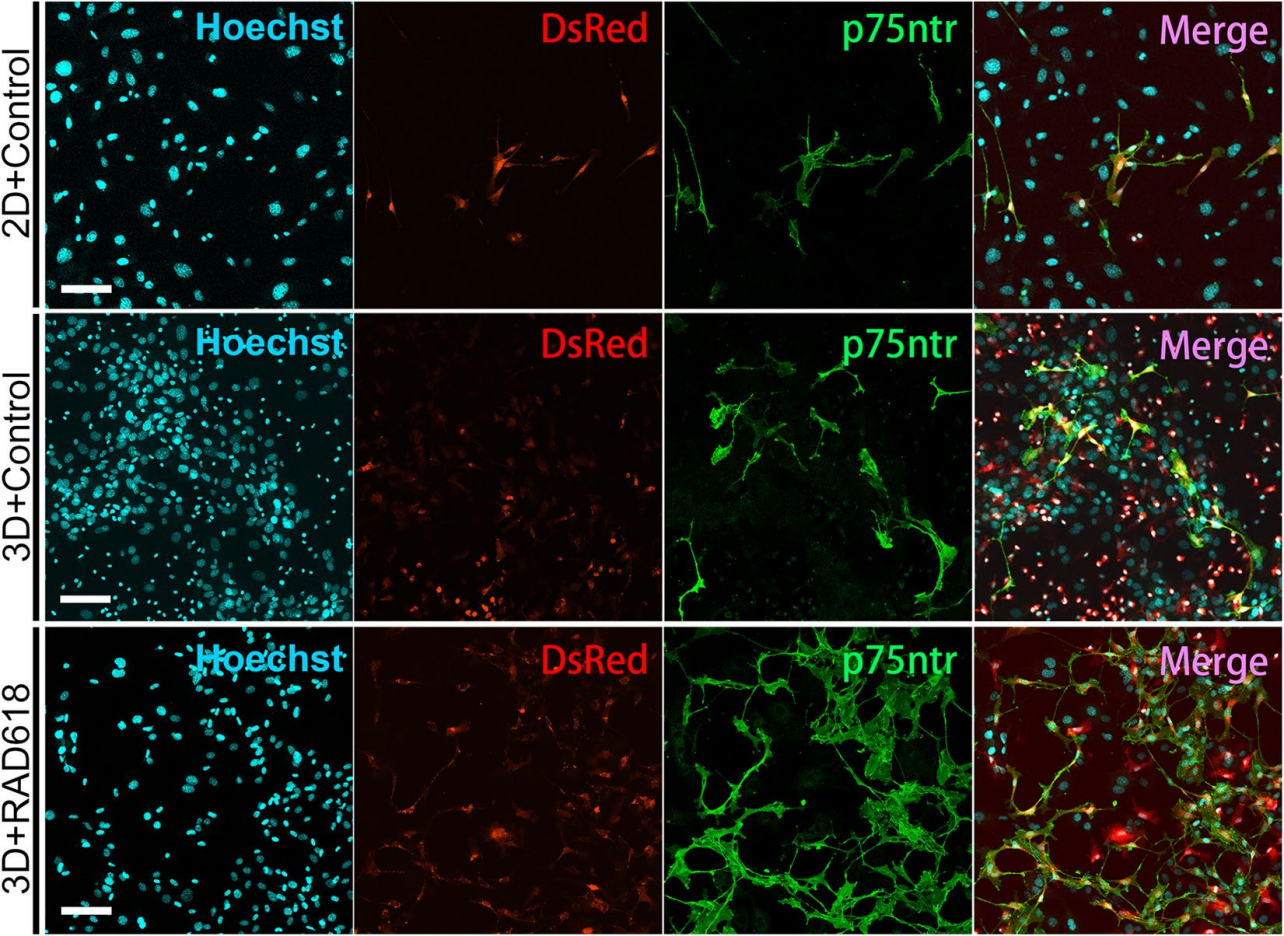
RAD618 supports continued expression of p75NTR in long-term primary OEC cultures. Panel 1: Hoechst (nuclear stain, aqua). Panel 2: DsRed-expressing OECs (red) Panel 3: p75NTR immunolabelling (green). Panel 4: merged images showing co-localisation of DsRed and p75NTR. *Top row:* Primary mouse OECs (DsRed) cultured for 14 days in 2D prior to spheroid formation. *Middle and bottom row:* Long-term 3D cultures of primary OECs; shown are cells which had migrated out of the spheroids after 55 days in culture in the absence (middle row) and presence of 2 µM RAD618 (bottom row). Scale bar: 100 µm.

We assessed the effects of long-term RAD618 treatment on p75NTR expression by primary OECs in long-term culture. We first fixed and immunolabelled OECs for the p75NTR immunolabelling prior to spheroid formation and showed that p75NTR co-localized with DsRed fluorescence (Fig. 4, top row). We then assessed expression of p75NTR in OECs which had migrated out of 3D cell spheroids after 55 days in culture, in the absence and presence of RAD618 (Fig. 4, middle and bottom row). DsRed-expressing OECs in the RAD618-treated groups, which had proliferated more than in the control group, expressed the p75NTR (at varying levels as previously described^19^). This result demonstrates that RAD618 stimulates proliferation of OECs, which continue to express p75NTR in long-term culture.

### RAD618 induces morphological changes in OECs

**Figure 5:**
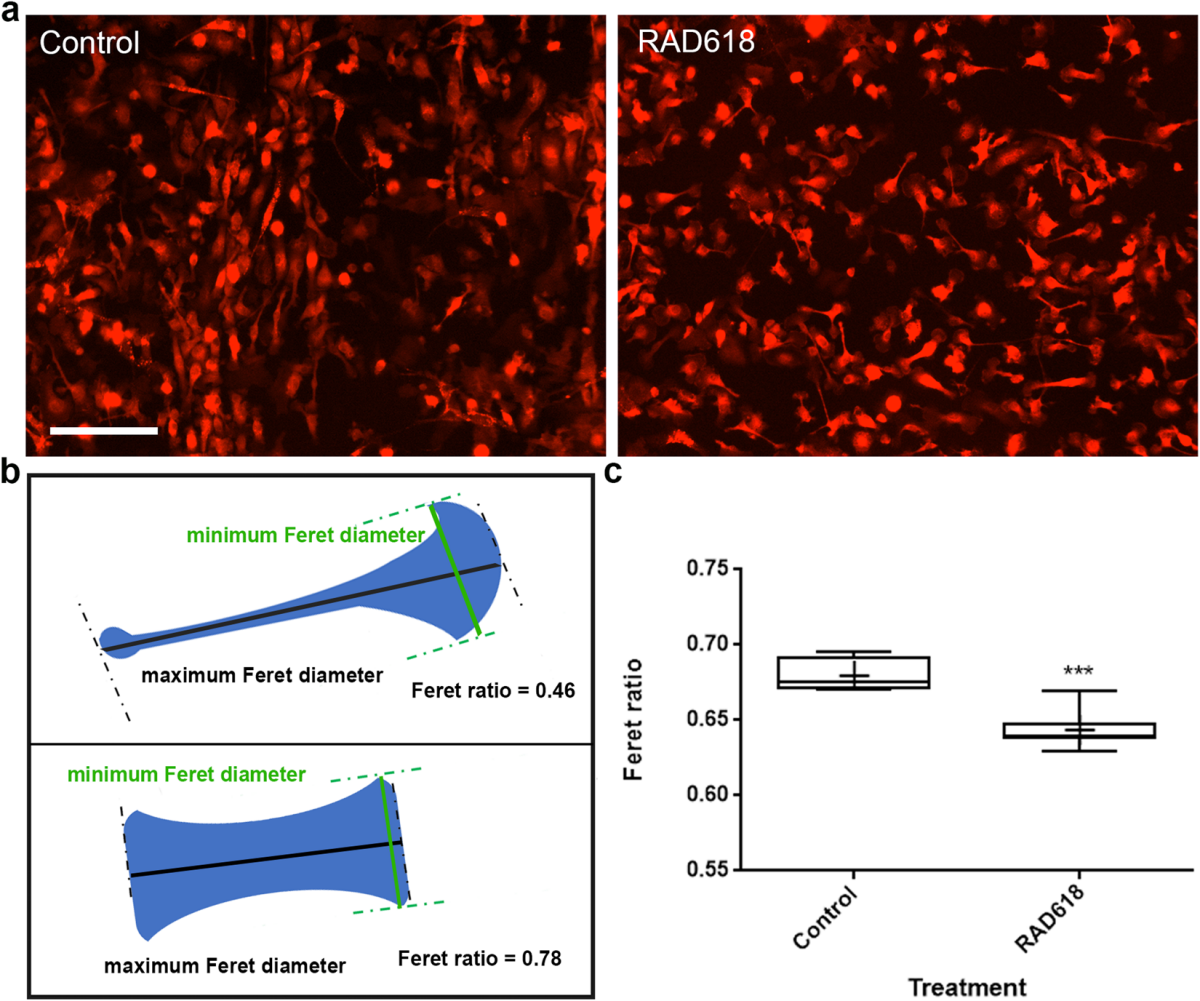
OEC morphology changes induced by RAD618 treatment. The morphology of live cells was analyzed after 30 days of incubation in medium containing RAD618 (2 µM) or in control medium. (**a**) Representative images of primary mouse OECs (DsRed fluorescence) incubated in control medium or with RAD618 at day 30 in culture. Scale bar: 100 µm. (**b**) Slender, bipolar cells exhibit a low value of Feret ratio (minimum Feret diameter/maximum Feret diameter) compared to round or flattened cells. Image created using CellProfiler 3.0 software (cellprofiler.org). (**c**) Cells incubated with RAD618 had a significantly lower value of Feret ratio than cells in control medium. The CellProfiler software was used to automatically select and measure the minimum and maximum Feret diameter of >3900 cells for control and >15,000 for RAD618 treatment. P < 0.001, Student’s t-test whiskers show range (lowest to highest Feret ratio).

Natural compounds such as curcumin can induce morphological changes in OECs, which correlates with increased migration and phagocytosis^13^. We imaged live primary mouse OECs over time in culture (using the IncuCyte system, in which cells are time-lapse imaged within an incubator). After 30 days in culture, we observed many flattened cells in the control group and, in contrast, a high proportion of bipolar cells with axial lamellipodia (lamellipodia localized at the leading edges of the cells) in the RAD618 group (Fig. 5a). To quantify this morphological change, we analyzed a series of cytoplasm morphology measurements using automated software (CellProfiler 3.0): form factor, solidity, eccentricity and Feret diameter ratio. We found that RAD618 only affected one of these parameters, the Feret diameter ratio. The Feret diameter is a measurement of the cell length/width projected in a specific direction, and the Feret ratio is the ratio between the maximum and minimum Feret diameter (Fig. 5b). A bipolar cell has a lower Feret ratio than a round cell, and thus, this method can be used to assess the level of polarization (bipolarity) in cells^46^.

The cells in the RAD618 treatment group had a significantly lower value of Feret ratio comparing to control group (Fig. 5c) and were thus more bipolar. Thus, RAD618 treatment promotes a bipolar morphology of OECs cells, which typically exhibits axial lamellipodia. Bipolarity of OECs may be correlated with increased migratory capacities; bipolar OECs have been shown to migrate ~3-fold faster than flattened OECs^47,48^.

### RAD618 incubation results in slowed OEC migration, most likely due to increased cell division frequency

**Figure 6:**
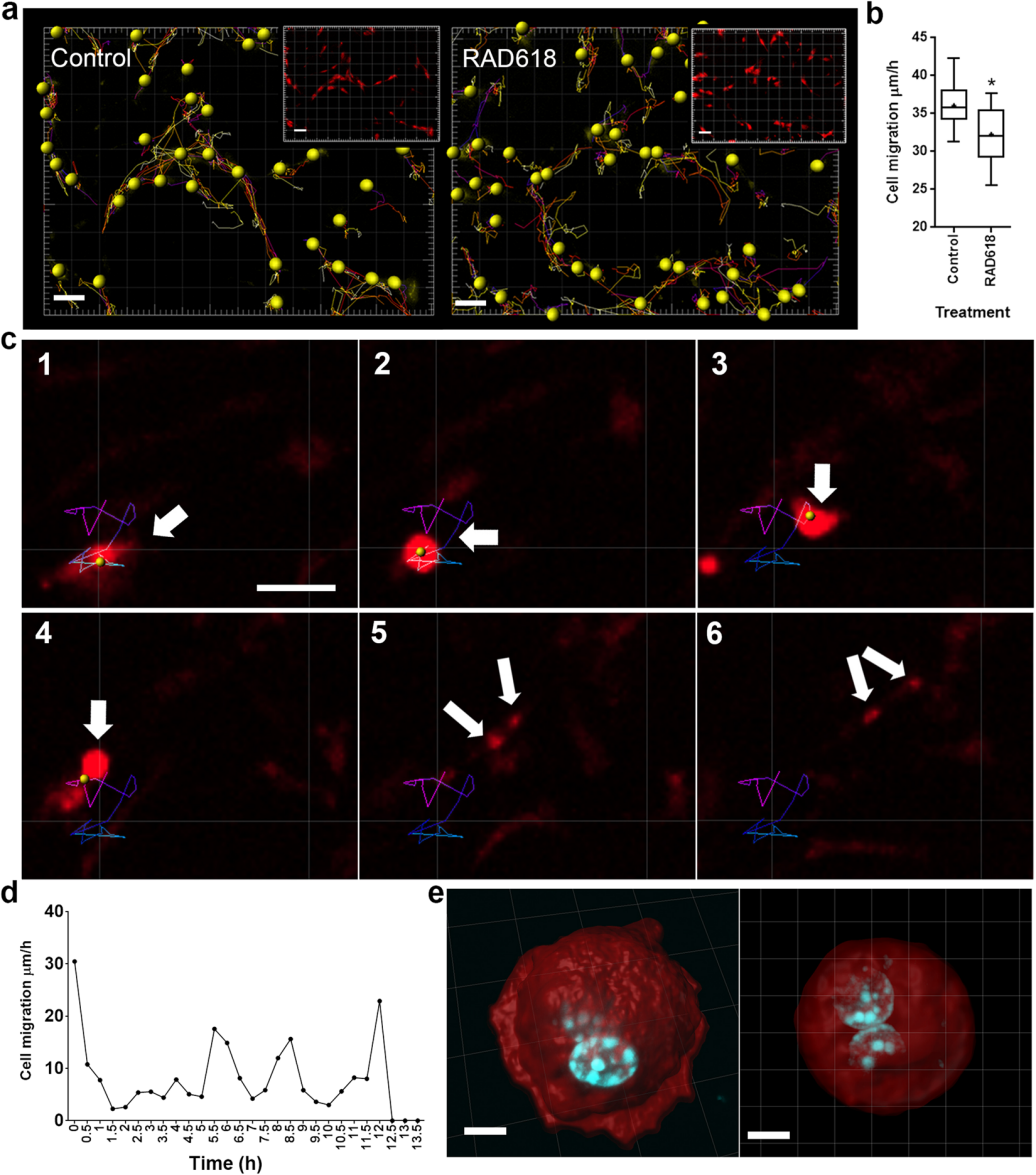
OEC migration rate is decreased by RAD618, most likely due to increased cell division frequency. Primary OECs were incubated in the absence or presence of RAD618 for 7 days. Then, cell migration was visualised and analyzed over 24 h using Imaris 7.4.2 software (imaris.oxinst.com). (**a**) Migration track of cells generated by Imaris software in the control and the RAD618-treated groups. Scale bar: 100 µm. (**b**) Migration rate of OECs in the two groups. n = 3 repeats × 755 cells; p < 0.05, Student’s t-test, whiskers show minimum to maximum migration rate. (**c**) The migration track of a cell before division. Scale bar: 20 µm. (**d**) The cell migration rate at different time points before cell division. (**e**) The 3D reconstruction of cells in telophase during mitosis under RAD618 treatment. Scale bar: 5 µm.

Because of the effects on RAD618 on OEC morphology, we hypothesized that RAD618 may promote migration. We tested this by tracking migration of individual primary mouse OECs imaged using the IncuCyte live cell system. OECs were incubated in control medium or in medium containing RAD618 for one week. Migration rate was assessed on day 7 in culture; images were captured every 30 min and the tracking feature in the Imaris software was used to determine total migration distance (converted to migration rate) per cell. Examples of movement tracks of OECs incubated in the absence or presence of RAD618 are shown in Fig. 6a. The average migration rate of OECs in RAD618-containing medium (32.24 µm/h) was significantly slower than in control medium (36.04 µm/h), contrary to our hypothesis (Fig. 6b). However, by identifying dividing cells in the recordings, and analyzing how their migration rate changed before and during division, we found that migration slowed down immediately before cell division (Fig. 6c,d). The average migration rate was only 7.93 µm/h before cell division, and there was a static period of approximately 90 min in the horizontal direction just before division. Thus, since RAD618 promotes cell proliferation, the average slowed migration rate may be due to increased cell division frequency during the imaged period. Figure 6e shows the OECs in a rounded-up shape containing two nuclei just before cell division.

### RAD618 modulates aspects of OEC phagocytosis

**Figure 7:**
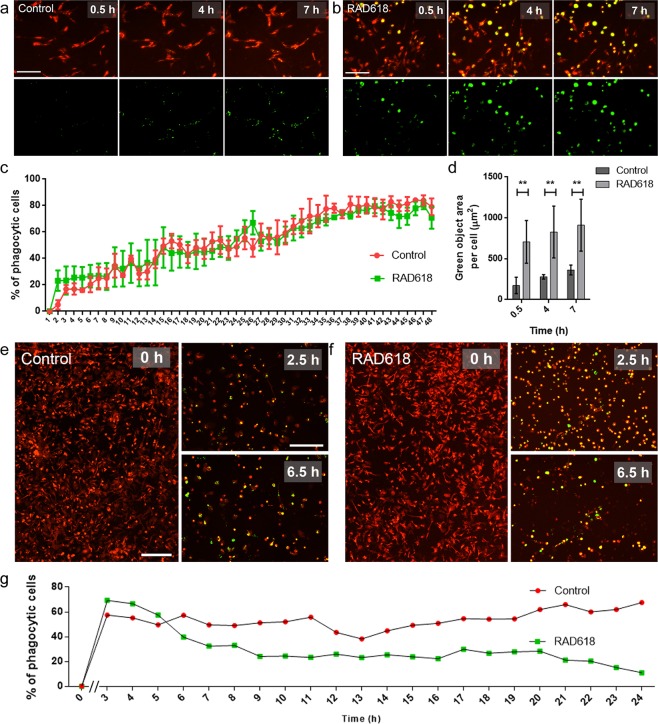
Effects of RAD618 on the phagocytic activity of primary mouse OECs. Shown are effects of RAD618 on the phagocytic activity after (**a**–**d**) 7 days pre-incubation and (**e**–**g**) 55 days pre-incubation (long-term assay). (**A**,**B**) Top panels show OECs (DsRed); bottom panels show green fluorescent BioParticles inside acidic organelles in cells at 0.5, 4 and 7 h after BioParticle addition. (**a**) Control cells. (**b**) Cells pre-treated with RAD618 (2 µM) for 7 days. (**c**) The percentages of OECs containing phagocytosed *S. aureus* bioparticles at different time points. (**d**) Green object area per cell (green *S. aureus* BioParticle area in individual DsRed OECs) was measured at 0.5, 4 and 7 h after BioParticle addition. Error bars show mean ± SEM. **p < 0.01, two-way ANOVA, Sidak’s multiple comparison test, 3 repeats. (**e**) mOECs (DsRed) with green fluorescent BioParticles inside acidic organelles at 0, 2.5 and 24 h in the control group. (**f**) OECs (DsRed) pre-treated for 55 days with RAD618 (2 µM) with green fluorescent BioParticles in cells at 0, 2.5 and 6.5 h. (**g**) The percentage of cells containing phagocytosed bioparticles at different time-points. Scale bars: 200 µm.

We then assessed whether RAD618 could stimulate OEC phagocytosis of pHrodo *S. aureus* BioParticles. These particles become fluorescent (green) at low pH, which occurs after the particles have been phagocytosed and internalized into acidic organelles such as lysosomes or phagosomes. OECs were pre-treated with RAD618 or control medium for one week, after which pHrodo-*S. aureus* BioParticles were added. Cells were imaged every 30 min to determine the percentages of cells with internalized particles over time, as well as the fluorescence intensity in individual cells at selected time-points. The assay was limited to 7 hours as degradation of the BioParticles can occur after this time leading to unreliable results. The percentages of phagocytic cells over time were not affected by RAD618 (Fig. 7a–d). However, at the 0.5 h, 4 h and 7 h time-points, the mean area of BioParticles inside of OEC was significantly higher in cells incubated with RAD618 than in control cells. This finding suggests that while medium-term pre-stimulation with RAD618 does not affect the percentage of phagocytic cells, it can increase the capacity for phagocytosis per cell.

To determine the effects of long-term pre-incubation with RAD618, primary mouse OECs were incubated with or without RAD618 for 55 days. Then, pHrodo *S. aureus* BioParticles were added to cell culture, and internalization of the BioParticles was assessed. The percentages of phagocytic cells in the RAD618 treatment group was significantly higher than in the control group at 2.5 h (Fig. 7e–g). From 6.5 h, the percentages of cells containing pHrodo-*S. aureus* BioParticles declined in the RAD618 treatment group (Fig. 7f,g), suggesting that the RAD618-stimulated cells had started degrading the BioParticles. Throughout the remainder of the assay (up to 24 h), the percentages of cells containing intracellular BioParticles was consistently lower in the RAD618 group (Fig. 7f,g) than in the control group (Fig. 7e,g). In fact, most cells in the control group still contained BioParticles at 12 h, while less than 20% of RAD618-treated cells contained BioParticles at this time-point (Fig. 7g). Thus, it appeared that long-term pre-incubation with RAD618 stimulated OEC phagocytosis, however, it should be noted that this assay was only performed once as repetitions were not feasible due to the very long incubation time.

## Discussion

One avenue for enhancing the therapeutic potential of OEC transplantation is using drugs to stimulate favourable cell behaviours. These include viability (to increase the number of cells available for transplantation and to promote cell survival after transplantation), migration (to enhance movement of cells into the injury site) and phagocytic activity (to promote clearance of cell debris). In a medium-throughput screen, we identified that the natural compound RAD618 (2-methoxy-1,4-naphthoquinone) could enhance the viability of an immortalized OEC cell line. We then investigated the mechanisms by which RAD618 exerted this effect. We showed that RAD618 altered the cell cycle by inducing cells to enter mitosis (to transit from S phase into G2/M phase). Furthermore, we identified a potential molecular target of RAD618, the transcription factor Nrf2, which regulates cellular defence responses, having an essential role in cellular redox homeostasis and proliferation. Nrf2 mediates these responses primarily via increasing the transcription of antioxidant response element (ARE genes)^49–51^. Nrf2 has also been demonstrated to specifically regulate cell cycle transition from G2 to M phase via the cyclin B-CDK1 complex^35^, consistent with the effects on OECs we observed in the current study. We also showed that a known Nrf2 agonist, the plant flavonol quercetin, stimulated OEC proliferation, confirming that stimulation of Nrf2 enhance proliferation of these cells. We have also previously shown that curcumin, another Nrf2 agonist, promotes proliferation of OECs^52^. Quercetin and curcumin have both been identified as Nrf2 agonists in different types of cells, including astrocytes and microglia^37–39,53–56^.

For transplantation into the injured spinal cord, OECs are isolated from the primary olfactory nervous system and then typically cultured *in vitro* for a long time prior to transplantation (2.5–10 weeks; typically ~ 4 weeks)^9,57,58^. For a compound to be relevant to the production process of OECs, it must therefore be demonstrated to act on primary OECs in long-term culture. We showed that RAD618 could stimulate the long-term viability of primary mouse OECs; RAD618 treatment resulted in a 4.1-fold increase in cell numbers compared to control after 55 days in culture. It is also essential that the cells do not de-differentiate in long-term culture. We found that cells treated with RAD618 continued to exhibit the fluorescent reporter marker DsRed (due to expression of the DsRed protein driven by the S100β promoter) and to express the OEC marker p75NTR in long-term cultures. Maintenance of p75NTR expression has previously been identified as a challenge in long-term OEC culture^42^. We also found that standard cell medium alone did not sufficiently support long-term OEC culture, suggesting that a stimulant is necessary to obtain a high number of healthy OECs for transplantation. Thus, RAD618 could potentially be incorporated into the *in vitro* production pipeline of OECs to increase the proliferation of the cells and thereby shorten the time from nasal biopsy to sufficient cells for transplantation.

While OECs are naturally migratory cells, capacity for migration into the injury site is variable^21–23^. We showed that RAD618 modulated OECs towards a bipolar morphology with large axial lamellipodia, a morphology previously shown to be associated with increased capacity for migration^47,48^. We therefore speculated that RAD618 might promote OEC migration, but we found that cells incubated with RAD618 migrated slower than control cells. We suggest that the reason for this is that RAD618 stimulates mitosis, as we showed that cells undergoing division stop migrating. It is also possible that the bipolar morphology alone is not associated with increased capacity for migration. We have previously shown that radial (not axial) lamellipodia are crucial for OEC migration^18^, and we did not observe any noticeable effect of RAD618 on radial lamellipodia.

We also found that RAD618 stimulated aspects of OEC phagocytosis. Pre-treatment of OECs with RAD618 for one week increased the phagocytic capacity by individual cells. Long-term pre-treatment (55 days) increased the percentage of phagocytic cells in the culture. It is possible that the morphology change induced by RAD618 (bipolar morphology, large axial lamellipodia) correlates with increased capacity for phagocytosis. We have previously shown that axial lamellipodia play an essential role in detecting material to be phagocytosed by Schwann cells^52^. Therefore, the axial lamellipodia may also be crucial for phagocytic activity in OECs. It is also possible that the effects on Nrf2 are connected to phagocytic ability. Nrf2 has been identified as an up-regulator of phagocytic activity in mouse macrophages^59^.

Napthoquinones are widespread in nature, occurring for example in actinomycetes, plants and fungi. These compounds exhibit a range of pharmacological properties ranging from anti-inflammatory, antibacterial, and antiviral properties to cytotoxic effects (Dictionary of Natural Products Database^45^). They are used traditionally for these properties in South American, Chinese, African and Indian medicines, and are interesting candidates for cancer treatments due to their cytotoxic effects^60,61^. 2-Methoxy-1,4-naphthoquinone (RAD618) has anti-tumour activities against human hepatocellular carcinoma cell line^62^ and U373 glioblastoma cells^63^. Of note, we did not detect any cytotoxic effects for RAD618 except at the highest concentration tested in our initial screen (10 µM). Indeed, RAD618 was shown to stimulate cell proliferation and maintenance of phenotype. Other effects reported for 2-methoxy-1,4-naphthoquinone include antipruritic activity^29^. However, due to the reactive 1,4-benzoquinone moiety found within napthoquinones, this particular structure class has been given a PAINS (Pan Assay INtereference compoundS) classification^64^ and thus some caution needs to be taken when considering further therapeutic development. Interestingly, other studies have shown that napthoquinones can have cytoprotective effects, in particular in the central nervous system, where they have been shown to protect against oxidative damage and neuroinflammation. They can pass the blood-brain barrier (BBB), and have been suggested as drug leads for ischemic stroke and Alzheimer’s disease^65–72^. The fact that these compounds can pass the BBB makes it possible that they could be used not only to pre-stimulate OECs prior to transplantation, but also to induce sustained potentially favourable effects over time. To date, little is known regarding their direct effects on glial cells, but their effect on neuroinflammation is thought to involve modulation of glia^73–75^.

Here, long-term treatment of OECs with RAD618 was shown to promote cell proliferation and phagocytosis. This finding is relevant from a therapeutic perspective; ideally, a drug used to promote favourable cell behaviours in OECs would be given prior to transplantation as a pre-treatment, rather than administered into the injury site. Thus, RAD618 may be used to pre-condition OECs towards better repair. The effects of RAD618 on cell proliferation can make the expansion of OECs on large scale possible, greatly reducing the time-period of OEC preparation prior to transplantation. Whilst RAD618 was also shown to promote other OEC behaviours associated with neural repair, the compound is most likely to be useful *in vitro* due to its limited drug-like properties. However, the fact that RAD618 also promoted OEC migration and phagocytic activity confirms that it is possible to stimulate these properties in OECs, and paves the way for further drug screening to identify compounds with similar activity but better drug-like properties.

## Methods

### Cell culture

#### GFP-mOECs

Immortalized mouse OECs expressing green fluorescent protein (GFP) (GFP-mOECs)^13^ were gifted from Prof. Filip Lim (Universidad Autónoma de Madrid, Spain). GFP-mOECs had initially been obtained from primary cultures of olfactory bulb OECs from GFP-expressing mice^76,77^. GFP-mOECs were cultured in DMEM/F12 supplemented with 10% fetal bovine serum (FBS) and 50 µg/mL gentamicin.

#### Primary mouse OECs

Primary OECs were isolated from the lamina propria of the nasal mucosa from S100ß-DsRed transgenic mice as previously described^18^. Primary OECs were maintained in DMEM supplemented with 10% FBS, 1% glutamax and 50 µg/mL gentamicin from 5 to 10 days before compound testing. These primary cultures contain both DsRed-expressing OECs and other cell types not expressing DsRed (mainly fibroblasts)^18^.

All experiments involving animals and transgenically modified cells were conducted with the approval of Griffith University’s University Biosafety Committee (approval NLRD/09/15_Var7) and Animal Ethics Committee (approval GRIDD/03/18/AEC) and in accordance with the guidelines of the National Health and Medical Research Council of Australia and the Australian Commonwealth Office of the Gene Technology Regulator.

### Open access compound library, microtitre plate preparation and hit compound resupply

The Davis open access natural product-based library consists of 472 distinct compounds, the majority (53%) of which are natural products that have been obtained from Australian natural sources, such as endophytic fungi^78^, plants^79^, macrofungi^80^, and marine invertebrates^81^. Approximately 28% of this library contains semi-synthetic natural product analogues^82^, while a smaller percentage (19%) are known commercial drugs or synthetic compounds inspired by natural products. The Davis library housed within Compounds Australia (www.compoundsaustralia.com), was dispensed into microtiter plates as 5 mM solutions in DMSO. Library compounds were either isolated in quantities ranging from 0.2 mg to > 50 mg or purchased from commercial suppliers. The natural product isolation procedures or semi-synthetic studies for the majority of compounds in this unique library have been previously published^78–81^. All compounds were > 95% pure when submitted for storage within Compounds Australia.

The hit compound (RAD618 = 2-methoxy-1,4-naphthoquinone) identified from this library was sourced from a commercial supplier (Sigma Aldrich, Cat. No. #189162, 98%). Fresh RAD618 from a dry-stored commercial stock was provided following initial screening for reconfirmation testing and further biological evaluations. Briefly, the hit compound 2-hydroxy-1,4-naphthoquinone was dissolved in 100% DMSO to make up a fresh 5 mM stock solution. RAD618 was then diluted in standard culture medium (final concentration consistently being 0.2% DMSO) and added to GFP-mOECs cultures at 1 µM and to primary OECs cultures at 2 µM. The control condition was the standard culture medium containing 0.2% DMSO.

### GFP-mOEC viability assay

A resazurin assay was used for determining cell viability. Viable cells metabolize the weakly fluorescent resazurin to resofurin, which exhibits strong red fluorescence (585 nm emission wavelength) (Anoopkumar-Dukie *et al*., 2005; Zhang, Du, & Zhang, 2004). Cells were incubated with resazurin at 50 µM (Sigma-Aldrich) in the absence (medium only vehicle control) or presence of RAD618 or (for one assay, Fig. 2d) the Nrf2 agonist quercetin (Sigma-Aldrich) for 4 h at 37 °C (5% CO_2_). The fluorescent signal was quantified with a plate reader (EnVision Multilabel) at 535/595 nm.

### GFP-mOEC cell cycle analysis

GFP-mOECs were seeded in 6-well plates at 100,000 cells/well. After 24 h, the medium was replaced by medium containing 1 µM RAD618. After 24 h, cells were fixed by 70% ethanol for 30 min at 4 °C. Cells were washed by phosphate-buffered saline (PBS) twice and treated by ribonuclease (RNAse) for 1 h at 100 µg/ml, 37 °C. Cells were then stained with propidium iodide (10 µg/ml) overnight; staining intensity correlates with cell cycle phase (Gap 1 (G1), synthesis (S), or Gap2/mitosis (G2/M) phase)^31^. These three populations were detected and quantified using a Guava easyCyte Flow Cytometer, reading at 605 nm. To determine effects of RAD618 on the percentages of cells in the early stages of mitosis, we used immunolabelling for histone H3 phosphorylated at serine 10 (Ser10); see *Immunolabelling*.

### Nrf2 inhibitor cell viability assay

GFP-mOECs were incubated with the Nrf2 inhibitor N-[4-[2,3-dihydro-1-(2-methylbenzoyl)-1H-indol-5-yl]-5-methyl-2-thiazolyl]-1,3 benzodioxole-5-acetamide (ML385, Sigma-Aldrich; stock solution in DMSO) at 5 µM for 24 h (final concentration of DMSO: 0.2%). Then, mOECs (in the presence/absence of ML385) were seeded onto 96-well plates at 6000 cells/well. The test groups were: (1) Control (mOEC in culture medium containing 0.2% DMSO), (2) mOECs with 1 µM RAD618, (3) mOECs which had been pre-incubated for 24 h with ML385 and (4) mOECs with 24 h ML385 pre-treatment + 1 µM RAD618. For the ML385 groups: cells were continuously exposed to ML385 also after re-seeding.

### Three-dimensional (3D) culture and purification of primary mouse OECs

When cultured in our 3D Naked Liquid Marble (NLM) system^40^, mixed cultures of primary OECs and contaminating cells spontaneously self-organize so that DsRed-expressing OECs naturally separate from other cells (DsRed(−); primarily fibroblasts). Thus, the NLM system can be utilized to separate and purify cell populations. OECs were cultured in NLMs as previously described^40^. Briefly, cells were cultured in droplets (NLMs) on a superhydrophobic plate; here, cells rapidly spontaneously form uniformly sized spheroids inside the NLM. Cell spheroids were transferred to 8-well chamber glass plates. When cells migrated out from spheroids, DsRed(+) OECs and DsRed(−) cells formed two distinctly separated groups, allowing selective ablation of DsRed(−) cells using laser microdissection. To determine optimal time in culture for removal of DsRed(−) cells, we first assessed the timing of migration for the two groups. Within the first two days, DsRed(−) cells migrated out the spheroid. At Day 3, DsRed(+) OECs started emerging from spheroids. At Day 4, DsRed(−) cells continued to expand outward. DsRed(+) OECs and DsRed(−) cells were mixed. Therefore, there was a 48 h delay of DsRed(+) OECs migration out the spheroid compared to DsRed(−) cells (Supplementary Fig. 1a). We therefore selected the 0–48 h time-window for removal of DsRed(−) cells. At 24, 32 and 40 h, DsRed(−) cells were ablated using a MMI CellCut Laser Microdissection System (Supplementary Figure 1b). The laser microdissection was operated under a 20x objective lens on the surface of each well. Using a high pulse rate and low voltage UV-laser, the accuracy of this instrument reaches 0.3 µm. We first tested the effects of this purification step on the final purity of OEC 3D cultures over 30 days prior to using this method for proliferation assays. Olympus IX73, Olympus FV1000/3000 Confocal and Nikon Eclipse Ti2 were used for imaging the cells. 3D reconstruction was generated and analyzed by Imaris software. We found that using laser cutting increased the purity of DsRed(+) OECs from 32 to 44% (Supplementary Fig. 2).

### Long-term proliferation assays for primary OECs

Primary mouse OECs cultured and enriched as described above were incubated in the absence (control) and presence of RAD618 for 55 days, during which cells were imaged using an IncuCyte S3 (Sartorius) live cell imaging system to enable time-lapse imaging of live cells within a standard incubator. Time-lapse images were captured under a 20x objective at 30 min intervals. Bright-field imaging was used to detect all cells, and fluorescence imaging (excitation peak around 558 nm) was used to detect DsRed-expressing OECs. After 55 days, cells were detached by TrypLE reagents (Gibco) and counted by a Countess II FL Automated Cell Counter and in a hemocytometer.

### Cell morphology and migration analysis (primary OECs)

#### Morphology

Cells were imaged using the IncuCyte system as described above. A live-cell morphology analysis system was built by CellProfiler 3.1.8 (free open-source software). The cytoplasm (primary objects) was recognized by *Global* (threshold strategy). The thresholding method was *Otsu*, the threshold smoothing scale was 1.3488 and the threshold correction factor was 1.2. The intensity value was used for distinguishing clumped cytoplasm (primary objects). The *form factor*, *solidity*, *eccentricity* and *Feret diamete*r of the cytoplasm were measured. The form factor (4*π*area/perimeter^2^) measures the cell shape, with a circle having the largest area to perimeter ratio, approaching a value of 1. Solidity (cytoplasm area/convex hull area) measures the proportion of pixels in the convex hull of a cell that are also within the cytoplasm, giving an indication of how ruffled the cell border is with a value approaching 1 suggesting that the cell more solid than ruffled. Eccentricity (distance between foci of an ellipse/distance along its major axis length) also gives an indication of cell circularity, with a circular cell having a ratio of zero and an elliptical cell having a ratio approaching 1. Feret diameter is the distance between two parallel lines tangent on either side of an object oriented in a specific direction. The ratio of the minimum Feret diameter to maximum Feret diameter is thus a measurement of bipolarity of the cells (see Fig. 5).

#### Migration

Live-cell images were captured every 30 min. Cell migration was tracked using the tracking function in Imaris 7.4.2 software (imaris.oxinst.com). Migration speed, track duration, track length, and track straightness were analyzed.

### Phagocytosis assays in primary mouse OECs

Uptake of pHrodo-labelled *Staphylococcus aureus* BioParticles (ThermoFisher) was used for measuring phagocytic activity of cells. pHrodo is pH-sensitive, allowing the BioParticles to only emit a fluorescent signal (green) when they enter the phagosome, in which the pH is low (acidic). Primary mOECs were treated with RAD618 (or control medium) for one week (medium-term) or 55 days (long-term). Then, the pHrodo *S. aureus* BioParticles were added to the culture. The IncuCyte live cell imaging system was used for recording time-lapse movies (20x objective and 30 min imaging intervals). *Percentages of phagocytic cells:* Co-localization of primary OECs (which express the fluorescent protein DsRed) with the pHrodo *S. aureus* BioParticles (green fluorescence signal) indicated that the cells had phagocytosed the particles. *Area of fluorescent BioParticles in cells:* The CellProfiler software (cellprofiler.org) was used to determine the area of the colocalized region as a measurement of the phagocytic activity in the medium-term assays.

### Cell staining and immunolabelling

Cells were fixed with 4% methanol-free formaldehyde in 1 × PBS for 15 min and permeabilized with 0.3% Triton X-100 for 15 min at room temperature (RT). Then, cells were incubated with 1% BSA blocking buffer for 1 h at RT. 1 x PBS was used for washing buffer.

#### Phosphorylated histone H3 in mOECs (cell cycle analysis)

Cells were incubated with rabbit anti-Ser10 Histone H3 (1:500, Labviva, ab5176) for 3 h at RT. The primary antibody was detected with donkey anti-rabbit Alexa 647 (1:500, Abcam, ab150075) (applied for 1 h at RT).

#### P75 neurotrophin receptor (p75NTR) expression in primary mouse OECs

Primary mouse OECs were first examined in conventional two-dimensional (2D) culture for co-localization of p75NTR and DsRed prior to spheroid formation. After 14 d in culture, cells were fixed and immunolabelled for p75NTR. Cell spheroids were then cultured as described above. Cells that had migrated out of spheroids after 55 d in culture were fixed and immunolabelled for p75NTR. Fixed cells or sectioned cell spheroids were incubated with rabbit anti-p75NTR antibody (1:200, BioLegend, 839701) for 3 h at RT, then detected with donkey anti-rabbit Alexa488 (1:500, Abcam, ab150073; 1 h at RT). Nuclei and cytoplasm were stained with Hoechst 33342 (1:2000, Thermo Fisher) and CellMask (1:2000, Thermo Fisher), respectively.

### Software and statistical analysis

Imaris 7.4.2 software (imaris.oxinst.com) was used to generate 3D reconstructions and for tracking of cell migration. CellProfiler 3.0 software (cellprofiler.org) was used for graphical analysis. GraphPad Prism 6 software was used for statistical analyses. Data distribution was assessed by the Kolmogorov–Smirnov test. Student’s t-test or analysis of variance (ANOVA) were used for determining statistical significance.

## Supplementary information

Supplementary Information.

Supplementary Information2.

Supplementary Information3.
