# Supplementary figures and images for: The plant natural product 2-methoxy-1,4-naphthoquinone stimulates therapeutic neural repair properties of olfactory ensheathing cells
Source: Sci Rep. 2020 Jan 22;10:951. doi: 10.1038/s41598-020-57793-2 (PMC6976649; doi:10.1038/s41598-020-57793-2)

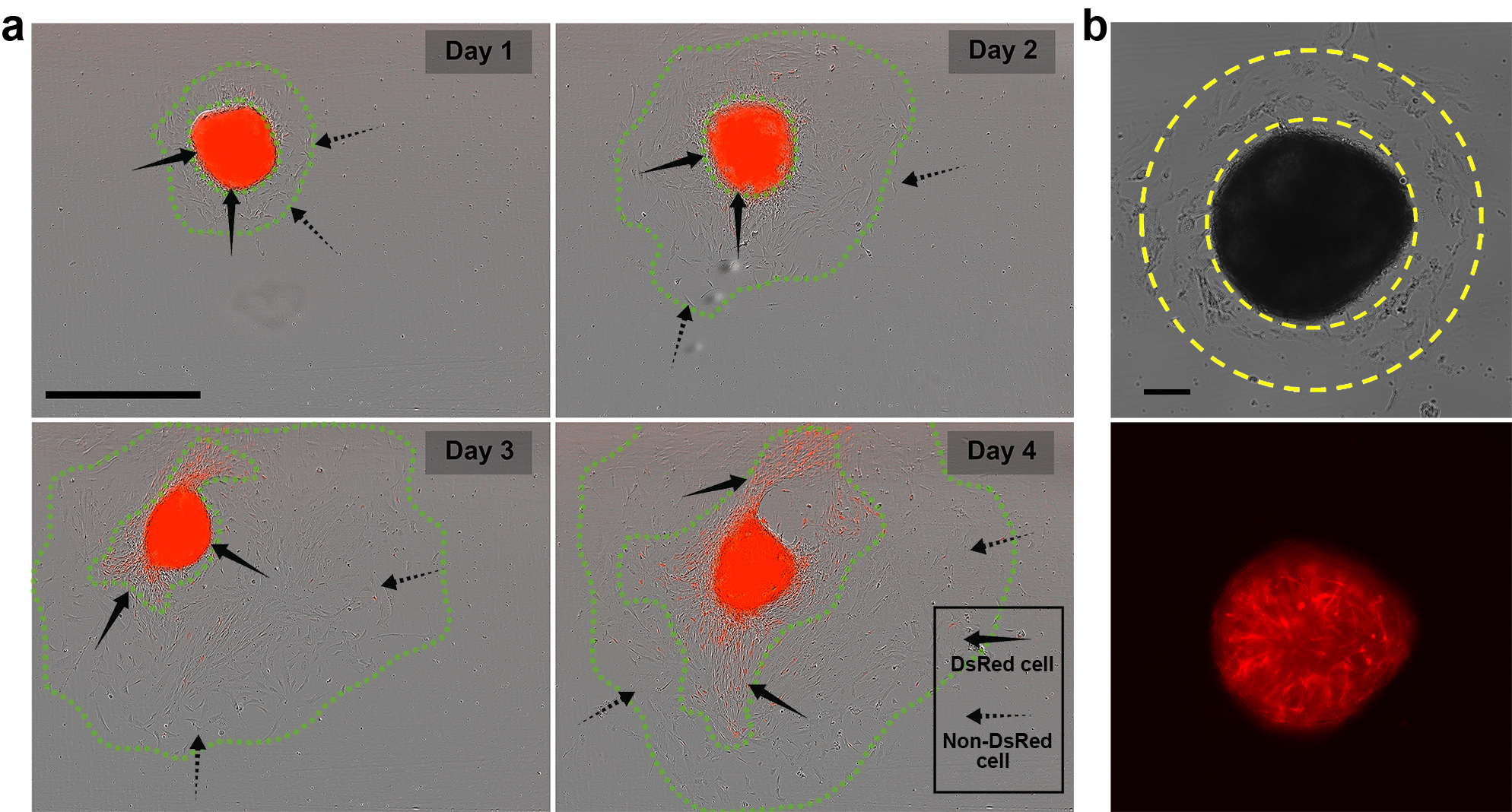

Supplement: Supplementary file 1 — Supplementary Information. [file 41598_2020_57793_MOESM1_ESM.tif]

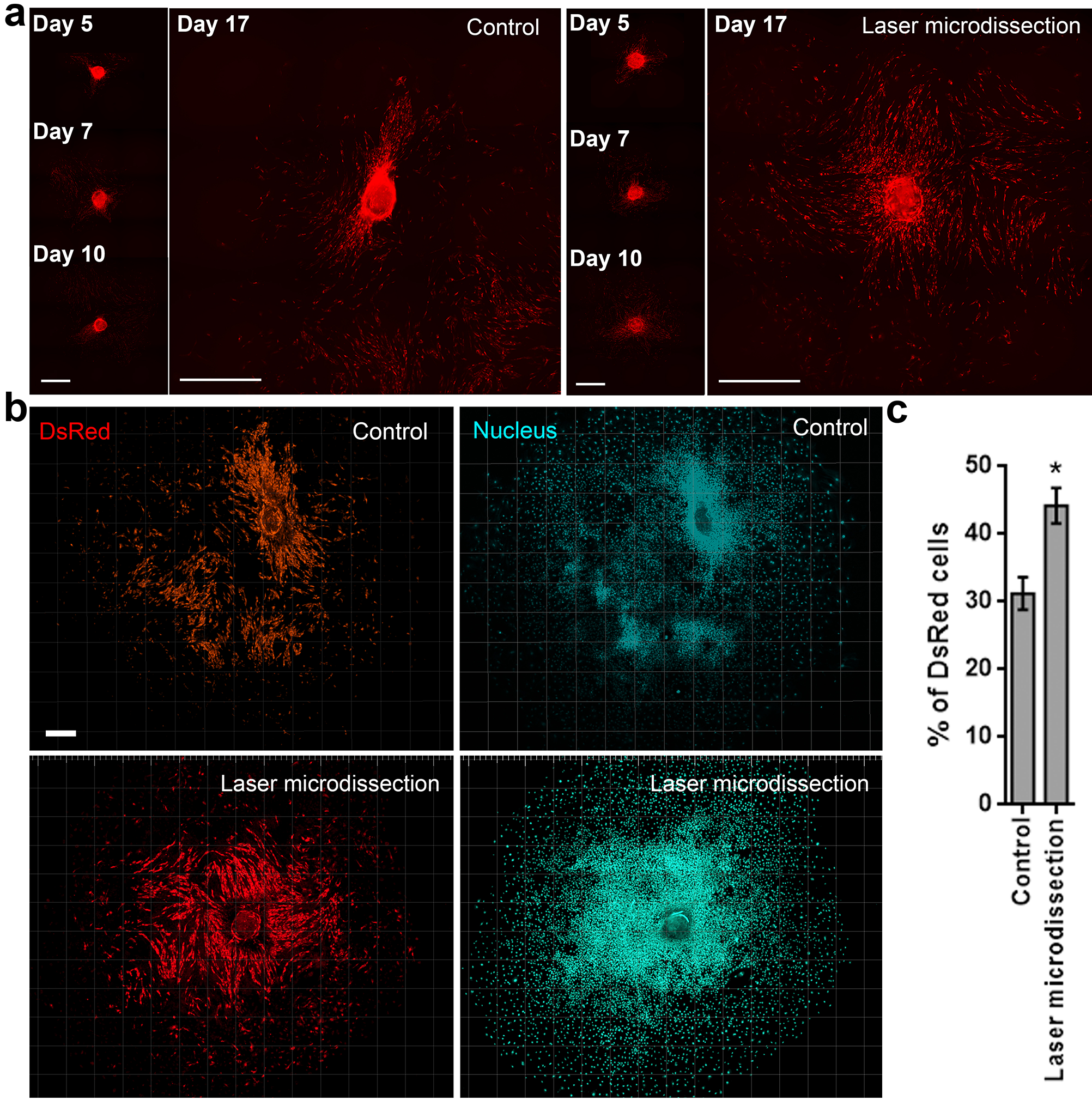

Supplement: Supplementary file 2 — Supplementary Information2. [file 41598_2020_57793_MOESM2_ESM.tif]
