# Supplementary material for: The plant natural product 2-methoxy-1,4-naphthoquinone stimulates therapeutic neural repair properties of olfactory ensheathing cells
Source: Sci Rep. 2020 Jan 22;10:951. doi: 10.1038/s41598-020-57793-2 (PMC6976649; doi:10.1038/s41598-020-57793-2)
Supplement: Supplementary file 3 — Supplementary Information3. [file 41598_2020_57793_MOESM3_ESM.docx]

**The plant natural product 2-methoxy-1,4-naphthoquinone stimulates therapeutic neural repair properties of olfactory ensheathing cells**

Chen M^1,2,3^, Vial ML^1,2,3^, Gee L^1,3^, Davis RA^2^, St John JA^1,2,3,#^, Ekberg JAK^1,2,3,#,*^

^1^Clem Jones Centre for Neurobiology and Stem Cell Research, Griffith University, Australia

^2^Griffith Institute for Drug Discovery, Griffith University, Nathan, QLD 4111, Australia

^3^Menzies Health Institute Queensland, Griffith University, Southport, QLD 4222, Australia

#These authors contributed equally

*Corresponding author: j.ekberg@griffith.edu.au

**Supplementary Figure 1.** **Enrichment of primary OEC cultures using 3D culture and laser microdissection**. (A) Images show cell migration from spheroids that have not been microdissected. The cultures contain OECs (DsRed) and other cell types not expressing DsRed. Cells not expressing DsRed migrate out during the first 48 h, while OECs primarily migrate out after this time-point. Scale bar, 800 µm. (B) Example of microdissected spheroid. After spheroid formation, the spheroids were transferred to a 2D culture plate. After 24 h in culture, cells not expressing DsRed were selectively ablated. Top image: brightfield. Bottom image: DsRed fluorescence.

**Supplementary Figure 2.** **The proportion of DsRed cells increased after laser microdissection.** After 30 days incubation post laser ablation, cells were fixed and stained for nuclei (Hoechst). (A) Shown are example images of cells migrating out of cultures which have not been subjected to laser ablation (left) and in which cells not expressing DsRed had been ablated at 24 h post transfer to 2D culture plate (right). Scale bar: 1000 µm. (B) After 30 days, cultures that had been laser microdissection ablated showed more extensive DsRed expression and more nuclei compared to control cultures. (C) The proportion of DsRed cells in control and laser microdissection group were 32 % and 44 % respectively, *p*=0.0219. Error bars show mean ± SEM (n=3 spheroids each for control condition and laser microdissection condition; each spheroid was generated using cells from three S100β-DsRed mice). The analysis was performed using Imaris 7.42 (Oxford Instruments).
